# Preparation of Rotenone Derivatives and *in Vitro* Analysis of Their Antimalarial, Antileishmanial and Selective Cytotoxic Activities

**DOI:** 10.3390/molecules191118911

**Published:** 2014-11-18

**Authors:** Yulieth Upegui, Juan F. Gil, Wiston Quiñones, Fernando Torres, Gustavo Escobar, Sara M. Robledo, Fernando Echeverri

**Affiliations:** 1 PECET, Instituto de Investigaciones Médicas, Facultad de Medicina, Universidad de Antioquia, Calle 70 #52-21, Medellín 050010, Colombia; E-Mail: yulexa1@gmail.com; 2 Grupo Química Orgánica de Productos Naturales, Instituto de Química, Facultad de Ciencias Exactas y Naturales, Universidad de Antioquia, Calle 70 #52-21, Medellín 050010, Colombia; E-Mails: juanf.gil@udea.edu.co (J.F.G.); wiston.quinones@udea.edu.co (W.Q.); fernando.torres@udea.edu.co (F.T.); gustavo.escobar1@udea.edu.co (G.E.); 3 Center for Development of Products against Tropical Diseases-CIDEPRO, Medellín 050010, Colombia

**Keywords:** rotenone, chemical transformations, antiparasite, differential activity, cytotoxicity

## Abstract

Six derivatives of the known biopesticide rotenone were prepared by several chemical transformations. Rotenone and its derivatives showed differential *in vitro* antiparasitic activity and selective cytotoxicity. In general, compounds were more active against *Plasmodium falciparum* than *Leishmania panamensis*. Rotenone had an EC_50_ of 19.0 µM against *P. falciparum*, and 127.2 µM against *L. panamensis*. Although chemical transformation does not improve its biological profile against *P. falciparum,* three of its derivatives showed a significant level of action within an adequate range of activity with EC_50_ values < 50.0 µM. This antiplasmodial activity was not due to red blood cell hemolysis, since LC_50_ was >>400 µM. On the other hand, all derivatives displayed a non-specific cytotoxicity on several cell lines and primary human cell cultures.

## 1. Introduction

Malaria and cutaneous leishmaniasis (CL) are tropical diseases that still remain a public health problem because of their high morbidity and mortality rates in many countries around the world. Unfortunately, the development of resistance to insecticides by insect vectors or decreased drug sensitivity by parasites have worsened the problem in those countries where therapeutic alternatives have become limited [1]. The availability of new molecules for current pharmacological targets could help overcome the problem of resistance to current drugs [2]. Therefore, new molecules are needed, preferably with novel mechanisms of action.

The mitochondrion is an essential organ for the survival of organisms, including *Leishmania* and *Plasmodium* parasites, and is a pharmacological target that has not been very well explored [3,4]. In *Leishmania*, for example, the redox system is critical for the survival of parasites inside macrophages, where an excessive production of reactive oxygen species (ROS) and oxidative stress occur, killing parasites [5]. This mechanism of action suggests that substances that inhibit redox metabolism or promote oxidative stress could be promising drug candidates [6]. Rotenone is a natural substance used as a biopesticide that inhibits the mitochondrial I complex of the respiratory chain, inducing apoptosis through enhancing mitochondrial ROS production [7,8,9,10]. Although it is known that ROS plays an important role in intracellular parasite elimination, the antiparasite activity of rotenone or derivatives remains unknown.

On the other hand, one of the limiting factors for using natural products in the search of new lead molecules is their low availability for chemical transformations, determination of structure-activity relationships, molecular optimization and additional preclinical and clinical assays. Surprisingly, rotenone has not been thoroughly analyzed concerning its antiparasite potential in spite of being marketed in high quantities as a botanical insecticide. Additionally, its mechanism of action related to redox balance alteration in the mitochondrion offers a pharmacological target to be explored, since oxidative stress induces DNA fragmentation, lipid peroxidation and abnormal protein synthesis. All of these effects have been well described in Parkinson disease model tests with this substance [11,12].

In this work, six rotenone derivatives were obtained, and their differential cytotoxic and antiparasitic activities were determined by *in vitro* studies.

## 2. Results and Discussion

### 2.1. Rotenone and Synthesized Derivatives

**Scheme 1:**
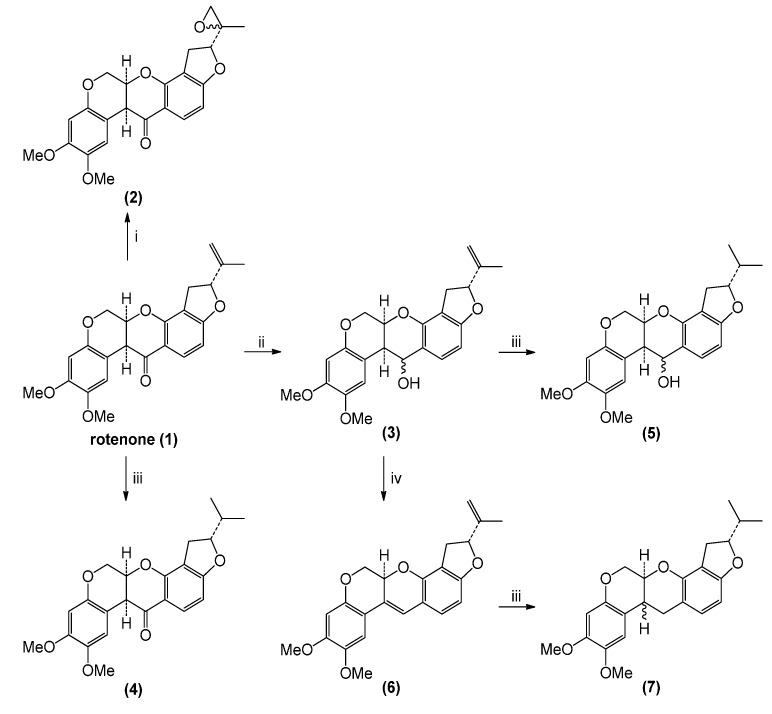
Preparation of rotenone derivatives.

Rotenone transformation reactions (Scheme 1) were directed toward two sites: the exocyclic double bond and the carbonyl group. In the case of epoxidation and reduction reactions, diastereomeric mixtures of reaction products resulted. Reaction yields were almost quantitative in all cases.

### 2.2. Cytotoxic and Antiparasitic Activities

The antiparasitic (antiplasmodial and antileishmanial) activities and cytotoxicity of the rotenone derivatives were evaluated following a previously reported method [13,14,15]. Antiparasite activity was reported as 50% effective concentration (EC_50_) values, while cytotoxicity was reported as 50% lethal concentration (LC_50_) values. Results are summarized in Table 1 and Table 2.

*Reagents and conditions*: i, 3-chloroperbenzoic acid (*m*-CPBA), dichloromethane (DCM), 0 °C. ii, NaBH_4_, MeOH, 0 °C. iii, H_2_, Pd/C, DCM. iv, HCl (dil.), CHCl_3_.

All derivatives showed differential and selective toxicity according to cell type, being more toxic on cell lines than primary cultures of human monocyte-derived macrophages (huMDM) and red blood cells (huRBC) (Table 1). Toxicity was not specific for a particular type of cell line, since it affected epithelial, renal, hepatic, neuronal and promonocytic cells.

*In vitro* assays of antiparasite activity (Table 2) displayed only moderate activity against *Plasmodium* and *Leishmania*. Rotenone has a CE_50_ 19.0 µM against *P. falciparum* and 127.2 µM against *L. panamensis*. Although chemical transformation apparently did not improve the biological activity, compounds **2**, **3**, **4** and **7** showed still a marginal level of action against *P. falciparum* with EC_50_ < 50 µM. Compounds **5** and **6** were essentially non-active (EC_50_ > 50 µM. The antiplasmodial activity was not due to the hemolysis of red cells, since the LC_50_ on huRBC was > 400 µM. In contrast, Compound **6** was 10-times more active than the parent molecule against *L. panamensis* (CE_50_ 127.2 µM *vs.* 12.7 µM, as shown in Table 2).

**Table 1:** *In vitro* cytotoxicity of rotenone and its derivatives*.* huMDM, human monocyte-derived macrophages.

Compound	Cytotoxicity (LC_50_, μM)						
	U937	CAD-R1	HepG2	Vero	huMDM	huRBC	
**1** (Rotenone)	115.1 ± 7.6	10.2 ± 0.0	1.8 ± 0.3	>508.0	378.8 ± 15.0	>508.0	
**2**	0.3 ± 0.1	29.0 ± 3.0	52.0 ± 6.3	81.3 ± 16.9	456.8 ± 22.7	>505.0	
**3**	0.1 ± 0.03	17.0 ± 3.4	1 2.0 ± 7.1	284.0 ± 23.6	518.3 ± 41.4	>523.0	
**4**	4.8 ± 2.5	185.9 ± 21.2	6.6 ± 3.8	>505.0	>505.0	>505.0	
**5**	35.2 ± 9.3	166.3 ± 20.1	18.6 ± 4.8	>503.0	>503.0	421.9 ± 33.2	
**6**	0.3 ± 0.1	50.5 ± 22.5	14.8 ± 2.9	68.0 ± 14.0	397.8 ± 43.4	>529.0	
**7**	0.7 ± 0.2	1.67 ± 0.9	5.0 ± 0.71	>476.0	>476.0	416.7 ± 76.9	
Amphotericin B	54.4 ± 0.1	54.5 ± 0.3	30.0 ± 2.2	15.6 ± 0.4	108.5 ± 10.0	19.4 ± 5.8	
Chloroquine	475.8 ± 16.3	2.2 ± 0.1	0.1 ± 0.03	26.0 ± 6.6	69.5 ± 11.3	>625.0	
Doxorubicin	0.1 ± 0.01	2.6 ± 0.7	0.7 ± 0.4	9.36 ± 2.2	1.29 ± 0.2	42.5 ± 16.9	

Data represent the median lethal concentration (LC_50_) mean value for each compound ± the standard deviation (SD) (*n* = 6 replicates) evaluated in the cell lines, U937, Vero, HepG2 and CAD-R1, and primary cultures of huMDM (macrophages) and huRBC (red blood cells).

**Table 2:** *In vitro* antiparasite activity of rotenone and its derivatives.

Compound	Biological activity CE_50_ (μM)	
	Leishmanicidal	Antiplasmodial
**1** (Rotenone)	127.2 ± 17.3	19.0 ± 8.6
**2**	>126.0	41.7 ± 12.6
**3**	>130.0	53.9 ± 2.4
**4**	>126.0	41.2 ± 13.4
**5**	>125.0	>50.0
**6**	12.7 ± 6.1	>53.0
**7**	>119.0	47.4 ± 0.2
Amphotericin B	0.8 ± 0.2	NT
Chloroquine	NT	0.06 ± 0.03

Data represent the median effective concentration (EC_50_) in μM for each compound ± SD (*n* = 6 replicates) in *L. panamensis* (intracellular amastigotes) and total forms of *P. falciparum*. NT: not tested, because it is not the control compound.

This effect could be related to changes in the polarity of the molecule. However, in comparison with the positive control chloroquine, the activity of this most potent of the current derivatives was about 300 times weaker which disqualifies compound **6** as an antimalarial.

Rotenone and derivatives **1**, **2**, **3** and **6** had an IS (which represents the ratio of cytotoxic LC_50_ over antiparasitic EC_50_, Table 3) > 8.0 in *P. falciparum*. In *L. panamensis*, for compound **6**, the IS was 31.3, and for rotenone, it was 3.1. For the other derivatives, the IS values were <4.0, which implies that the required dose to reach cytotoxic levels is almost three-times higher.

**Table 3:** Selectivity of rotenone and its derivatives (IS, index of selectivity).

Compound	Index of Selectivity	
	huMDM ^a^	huRBC ^b^
**1** (Rotenone)	3.1	>26.1
**2**	<3.6	>12.1
**3**	<3.9	>9.6
**4**	<4.0	>12.2
**5**	<4.0	<8.4
**6**	31.3	<9.9
**7**	<4.0	8.7
Amphotericin B	143.0	NC ^c^
Chloroquine	NC ^c^	>10.416

**^a^** IS LC_50_ in huMDM/EC_50_ for *L. panamensis*; **^b^** IS LC_50_ in huRBC/EC_50_ in *P. falciparum*; ^c^ NC: Not calculated, because neither the hemolytic activity of amphotericin B was determined on huRBC nor the cytotoxic activity of chloroquine was determined on huMDM.

## 3. Experimental Section

### 3.1. General Information

The solvents used for extraction and fractionation were distilled. For thin-layer chromatography, aluminum-backed F_254_ silica gel chromatoplates were used (Merck, Darmstadt, Germany). Column chromatographies were performed with silica gel G60 (Merck). ^1^H-NMR, ^13^C-NMR and two-dimensional spectra were obtained in a Bruker AMX 300 spectrometer operating at 300 MHz for ^1^H and 75.0 MHz for ^13^C using CDCl_3_ (Sigma-Aldrich, St. Louis, MO, USA) Shifts are reported in δ units (ppm) and coupling constants (*J*) in Hz. ELISA reader (Bio-Rad, Hercules, CA, USA).

RPMI-1640, DMSO, Ficoll-Hypaque, MTT, ethidium bromide, amphotericin B, sodium citrate and rotenone were obtained from Sigma-Aldrich (St. Louis, MO, USA). FBS, penicillin-streptomycin, DMEM, Triton X-100, Trypsin/EDTA were from Gibco (Grand Island, NY, USA). Proteinase K was from Promega (Madison, WI, USA). 

### 3.2. Chemical Transformations of Rotenone

*6aS,12aS-8,9-Dimethoxy-2-(2-methyloxiran-2-yl)-1,2,12,12a-tetrahydrochromen*o[3,4-b]*fur*o[2,3-h]*-chromen-6(6aH)-one* (rotenone epoxide, **2**): A mixture of rotenone (**1**, 200 mg, 0.50 mmol, 1 eq) and 3-chloroperbenzoic acid (*m-*CPBA, 368 mg, 2.1 mmol, 4 eq) in dichloromethane (DCM, 10 mL) was stirred at RT for 2 h; then, NaOH 2.0 M was added to the reaction mixture and extracted with DCM (2 × 15 mL). The combined organic layer was washed with brine solution (30 mL), dried (Na_2_SO_4_) and evaporated under reduced pressure. The residue was purified by chromatography on a silica gel column (hexane/EtOAc 4:1) yielding compound **2** (177 mg, 85%) as a colorless oil corresponding to a mixture of epoxides.^1^H-NMR (CDCl_3_) δ (ppm): 1.8 (3H, s), 2.1 (1H, s), 2.4 (1H, s), 2.9 (1H, dd, *J* = 8.1, 8.4 Hz), 3.3 (1H, dd, *J* = 8.1, 8.4 Hz), 3.8 (3H, s), 3.8 (1H, s), 3.9 (3H, s), 4.5 (2H, dd, *J* = 10.8 Hz), 4.7 (1H, dd, *J* = 10.8 Hz), 4.9 (1H, s), 5.3 (1H, t, *J* = 8.4 Hz), 6.4 (1H, d, *J* = 1.2 Hz), 6.8 (1H, d, *J* = 1.2 Hz), 6.6 (1H, d, *J* = 8.4 Hz), 7.3 (1H, d, *J* = 8.4 Hz). ^13^C-NMR (CDCl_3_) δ (ppm): 17.1 (CH_3_), 29.7 (CH_2_), 31.1 (CH_2_), 44.6 (CH), 55.8 (OCH_3_), 56.4 (OCH_3_), 63.8 (C), 67.5 (OCH_2_), 72.2 (CH), 88.0 (CH), 101.0 (CH), 104.9 (CH), 105.3 (C), 109.3 (CH), 112.7 (C), 113.2 (C), 130.1 (CH), 144.0 (C), 149.5 (C), 151.1 (C), 157.7 (C), 168.1 (C), 188.9 (C=O).

*2R,6aR,12aS-8,9-Dimethoxy-2-(prop-1-en-2-yl)-1,2,6,6a,12,12a-hexahydrochromeno*[3,4-b]*furo*[2,3-h]*chromen-6-ol* (rotenone alcohol, **3**). Rotenone (**1**, 400 mg, 1.0 mmol, 1 eq) in methanol (10 mL) was treated with NaBH_4_, (31 mg, 0.8 mmol, 0.8 eq) and stirred at 0 °C for 3 h; water was then added to the reaction mixture and extracted with DCM (2 × 15 mL). The combined organic layer was washed with brine solution (30 mL), dried (Na_2_SO_4_) and evaporated under reduced pressure. The residue was purified by chromatography on a silica gel column (hexane/EtOAc 4:1) yielding compound **3** as a light yellow solid (401 mg, 95%). Melting point (m.p.): 154–156 °C. IR (KBr): 3,445, 2,918, 2,865, 1,617. ^1^H-NMR (CDCl_3_) δ (ppm): 1.8 (3H, s), 2.9 (1H, dd, *J* = 8.1, 8.4 Hz), 3.3 (1H, dd, *J* = 8.1, 8.4 Hz), 3.8 (3H, s), 3.8 (3H, s), 4.1 (H, dd, *J* = 10.8 Hz), 4.6 (1H, dd, *J* = 10.8 Hz), 4.8 (1H, s), 4.9 (1H, s), 4.9 (1H, s), 5.2 (1H, s), 5.3 (1H, t, *J* = 8.4 Hz), 6.4 (1H, d, *J* = 1.2 Hz), 6.9 (1H, d, *J* = 1.2 Hz), 6.6 (1H, d, *J* = 8.4 Hz), 7.3 (1H, d, *J* = 8.4 Hz). ^13^C-NMR (CDCl_3_) δ (ppm): 17.1 (CH_3_), 32.0 (CH_2_), 38.1 (CH), 55.9 (OCH_3_), 56.4 (OCH_3_), 65.1 (OCH_2_), 66.4 (CH), 69.2 (CH), 86.6 (CH), 100.8 (CH), 102.8 (CH), 105.3 (C), 111.9 (CH), 112.1 (=CH_2_), 112.9 (C), 113.9 (C), 130.4 (CH), 143.8 (C), 143.9 (C), 149.5 (C), 151.1 (C), 149.7 (C), 161.9 (C).

*2R,12aS-8,9-Dimethoxy-2-(prop-1-en-2-yl)-1,2,12,12a-tetrahydrochromeno*[3,4-b]*furo*[2,3-h]*-chromene* (**4**). A mixture of rotenone (200 mg, 0.5 mmol, 1 eq) and palladium on carbon (10.0 mg, 10% mol Pd, 0.05 mmol) in DCM (5 mL) was treated with hydrogen and stirred at RT overnight. The reaction mixture was filtered through a pad of Celite, and the solvent was evaporated under reduced pressure. The residue was purified by chromatography on a silica gel column (hexane/EtOAc 4:1) yielding compound **4** as a white solid (195 mg, 98%). Melting point (m.p.): 204–206 °C. IR (KBr): 3,450, 2,960, 1,682, 1,610. ^1^H-NMR (CDCl_3_) δ (ppm): 1.1 (3H, d, *J* = 6.9 Hz), 1.1 (3H, d, *J* = 6.9 Hz), 2.0 (1H, m), 2.9 (1H, dd, *J* = 8.4, 8.7 Hz), 3.2 (1H, dd, *J* = 8.4, 8.7 Hz), 3.8 (3H, s), 3.8 (3H, s), 3.9 (1H, d, *J* = 3.3 Hz), 4.2 (2H, d, *J* = 12.0 Hz), 4.6 (2H, m), 4.9 (1H, m), 6.5 (2H, d, *J* = 7.2 Hz), 6.8 (1H, 2), 7.8 (1H, d, *J* = 7.2 Hz). ^13^C-NMR (CDCl_3_) δ (ppm): 17.6 (CH_3_), 17.9 (CH_3_), 29.4 (CH_2_), 33.2 (CH), 44.6 (CH), 55.8 (OCH_3_), 56.3 (OCH_3_), 66.3 (OCH_2_), 77.5 (CH), 90.8 (CH), 100.9 (CH), 104.8 (CH), 104.9 (C), 110.4 (CH), 113.1 (C), 113.4 (C), 129.8 (CH), 143.9 (C), 147.4 (C), 149.5 (C), 157.7 (C), 167.7 (C), 188.9 (C=O).

*2R,6aS,12aS-2-Isopropyl-8,9-dimethoxy-1,2,12,12a-tetrahydrochromeno-*[3,4-b]*furo*[2,3-h]*chromen-6(6aH)-one* (**5**). A mixture of compound **4** (200 mg, 0.5 mmol, 1 eq) and palladium on carbon (10.0 mg, 10% mol Pd, 0.05 mmol) in DCM (5 mL) was treated with hydrogen and stirred at RT overnight. The reaction mixture was filtered through a pad of Celite, and the solvent was evaporated under reduced pressure. The residue was purified by chromatography on a silica gel column (hexane/EtOAc 5:1) yielding compound **5** as a colorless oil (190 mg, 95%). IR (KBr): 3,436, 2,958, 1,709, 1,620. ^1^H- NMR (CDCl_3_) δ (ppm): 1.0 (3H, d, *J* = 6.6 Hz), 1.1 (3H, d, *J* = 6.6 Hz), 2.04 (1H, m), 2.9 (1H, dd, *J* = 6.6; 6.6 Hz), 3.1 (1H, dd, *J* = 6.6 ; 6.6 Hz), 3.4 (1H, m) 3.8 (3H, s), 3.9 (3H, s), 4.3 (1H, m), 4.6 (2H, m), 4.8 (1H, m), 4,9 (1H, m), 6.4 (1H, d, *J* = 8.1 Hz), 6,5 (1H, s), 6.7 (1H, s), 7.1 (1H, d, *J* = 8.1 Hz). ^13^C-NMR (CDCl_3_) δ (ppm): 17.7 (CH_3_), 18.2 (CH_3_), 30.1 (CH_2_), 33.3 (CH), 38.2 (CH), 55.9 (OCH_3_), 56.6 (OCH_3_), 65.1 (CH_2_) 66.39 (OCH_2_), 69.2 (CH), 89.5 (CH), 100.8 (CH), 102.7 (CH), 110.2 (C), 111.5 (CH), 111.7 (C), 115.7 (C), 130.3 (CH), 143.3 (C), 148.3 (C), 148.7(C), 149.7 (C), 160.52 (C).

*2R,6aR,12aS-2-Isopropyl-8,9-dimethoxy-1,2,6,6a,12,12a-hexahydrochromeno*[3,4-b]*furo*[2,3-h]*-chromen-6-ol* (**6**). This derivative was obtained by spontaneous decomposition of compound **4** after two days of exposure to daylight in chloroform. Once the solvent was evaporated under reduced pressure, a brown solid is produced. Melting point (m.p.): 167–169 °C. IR (KBr): 3,437, 2,924, 1,618, 1,508. ^1^H-NMR (CDCl_3_) δ (ppm): 1.8 (3H, s), 2.9 (1H, dd, *J* = 8.1, 8.4 Hz), 3.3 (1H, dd, *J* = 8.1, 8.4 Hz), 3.8 (3H, s), 3.9 (3H, s), 4.1 (1H, m), 4.6 (1H, m), 4.9 (1H, s), 5,1 (1H, s), 5.2 (1H, m), 5.3 (1H, m), 6.4 (2H, d, *J* = 8.1 Hz), 6.6 (1H. s), 6.9 (1H, d, *J* = 8.1 Hz), 7.0 (1H, s). ^13^C-NMR (CDCl_3_) δ (ppm): 17.2 (CH_3_), 31.6 (CH_2_), 55.9 (OCH_3_), 56.4 (OCH_3_), 67.9 (OCH_2_), 71.0 (CH), 86.6 (CH), 100.9 (CH), 102.8 (CH), 105.2 (CH), 110.8 (C), 112.1 (CH), 112.1 (=CH_2_), 112.8 (C), 116.8 (C), 123.2 (=C), 126.7 (=CH), 143.8 (C), 144.7 (=C), 149.1 (C), 149.4 (C), 150.3 (C), 161.3 (C).

*2R,6aS,12aS-2-Isopropyl-8,9-dimethoxy-1,2,6,6a,12,12a-hexahydrochromeno-*[3,4-b]*furo*[2,3-h]*-chromene* (**7**). Compound **6 ** (200 mg, 0.5 mmol, 1 eq) and palladium on carbon (Pd/C) (10.0 mg, 10% mol Pd, 0.05 mmol) in DCM (5 mL) Celite, and the solvent was evaporated under reduced pressure. The residue was purified by chromatography on a silica gel column (hexane/EtOAc 5:1) yielding compound **7** as a light yellow solid (190 mg, 95%). Melting point (m.p.): 136–138 °C. IR (KBr): 3,434, 2,927, 2,854, 1,679, 1,609. ^1^H-NMR (CDCl_3_) δ (ppm): 0.9 (3H, d, *J* = 3.0 Hz), 1.1 (3H, d, *J* = 3.0 Hz), 1.9 (1H, m), 2.8 (1H, m), 3.0 (1H, m), 3.1 (2H, m) 3.2 (1H, m) 3.8 (6H, s), 4.2 (2H, d), 4.5 (1H, m), 4.7 (1H, m), 6.3 (1H, d, *J* = 8.1 Hz), 6.4 (1H, s), 6.7 (1H, s), 6.8 (1H, d, *J* = 8.1 Hz). ^13^C-NMR (CDCl_3_) δ (ppm): 17.7 (CH_3_), 18.2 (CH_3_), 29.4 (CH_2_), 30.1 (CH_2_), 31.8 (CH), 33.2 (CH), 55.9 (OCH_3_), 56.6 (OCH_3_), 65.6 (OCH_2_), 68.7 (CH), 89.1 (CH), 100.6 (CH), 101.8 (CH), 111.2 (CH), 111.0 (C), 112.8 (C), 113.8 (C), 123.2 (CH), 143.8 (C), 147.8 (C), 149.4 (C), 150.3 (C), 160.1 (C).

### 3.3. Biological Activity Assays

The compounds were assayed *in vitro* for cytotoxicity on mammalian cells, antiplasmodial activity on asynchronized *P. falciparum* culture forms and leishmanicidal activity on intracellular amastigotes of *L. panamensis*.

#### 3.3.1. Cells and *in Vitro* Culture

U937 cells (CRL-1593^TM^) were isolated from human histiocytic lymphoma; Vero cells (CCL-81^TM^) were derived from the kidney of the African green monkey, *Cercopithecus aethiops*; HepG2 (HB-8065^TM^) cells were derived from human liver tissue, and CAD-R1 cells, a subclone, were derived from mouse neuronal cells [16]. All cell lines were from American Type Culture Collection (Manassas, VA, USA), except CAD-R1 cells that were kindly donated by D. Chikaraishi and J. Wang (Department of Neurobiology, Duke University Medical Center, Durham, NC, USA). All cell lines were cultured in standard conditions at 37 °C, 5% CO_2_. U-937 cells were cultivated in RPMI 1640 medium supplemented with 10% fetal bovine serum (FBS) and antibiotics (penicillin 100 U/mL and streptomycin 0.1 mg/mL). Vero, HepG2 and CAD-R1 were cultivated in DMEM medium supplemented with 5% FBS and antibiotics.

Primary cultures of human monocyte-derived macrophages (huMDMs) and human red blood cells (huRBCs) obtained from healthy donors after signing the informed consent were also used. The huMDM were obtained from peripheral mononuclear cells isolated by Ficoll-Hypaque 1077 gradient centrifugation. huMDMs were suspended at 3 × 10^5^ cells/mL in RPMI 1640 medium supplemented with 10% autologous serum. One mL of suspension was dispensed into each well of 24-well chamber slides and incubated at 37 °C, 5% of CO_2_ for 72 h, according to a previously described methodology [17,18]. On the other hand, huRBCs were obtained from sodium citrate anticoagulated whole blood. After centrifugation at 1,500 rpm for 10 min, the RBCs were recovered and suspended in RPMI 1640 medium at 50% hematocrit and stored at 4 °C until use.

#### 3.3.2. *In Vitro* Cytotoxic Activity

Cytotoxic activity of rotenone and it derivatives on U-937, Vero, HepG2, CD-R1 and huMDM cells was evaluated *in vitro* using the MTT (3-(4,5-dimethylthiazol-2-yl)-2,5-diphenyltetrazolium bromide assay) as described previously [13,14,15]. Cells were adjusted to 1 × 10^5^ U-937 cells/mL, 2.5 × 10^5^ Vero, HepG2 or CAD-R1 cells/mL and 5 × 10^5^ huMDM cells/mL in RPMI 1640 supplemented with 10% SBF and the corresponding concentration of each compound. Six serial-based four-dilution concentrations from 200 to 0.19 μg/mL were tested. The amount of formazan produced by the viable cells was registered as optical densities (OD) obtained in an ELISA reader (Bio-Rad) at 570 nm. Cells exposed to amphotericin B, doxorubicin and chloroquine were used as positive (cytotoxicity) controls, while unexposed cells were used as negative (viability) controls. Additionally, hemolytic activity on huRBCs was assessed by the osmotic lysis method [19]. In brief, O positive (O+) huRBCs were adjusted at 3% hematocrit saline solution and incubated in the presence of each compound (six serial four-fold dilutions from 200 to 0.19 µg/mL) at 37 °C. After 2 h, hemoglobin concentration in the supernatant was measured according to the OD obtained in an ELISA reader plate (Bio-Rad) at 570 nm. Cells exposed to saline solution with Triton X-100 were used as positive (lyses) controls, while unexposed cells were used as negative (viability) controls. Each compound and each concentration were assessed in triplicate in at least two separate experiments.

#### 3.3.3. *In Vitro* Antiplasmodial and Leishmanicidal Activities

The antiplasmodial activity of rotenone and its derivatives was evaluated on *Plasmodium falciparum* (chloroquine sensitive 3D7 strain) cultured in standard conditions [20]. The effect of compounds on the growth of the parasites was assessed by quantification of the parasite DNA stained with ethidium bromide (EtBr) [13]. Briefly, unsynchronized cultures of *P. falciparum* were adjusted to a 1.5%–2% parasitemia and 5% hematocrit in RPMI medium supplemented with 10% human serum. Then, 500 µL of parasites and 500 µL of each concentration of compound were placed in each well of 24-well plates. Plates were incubated for 72 h at 37 °C in a N_2_ (90%), CO_2_ (5%) and O_2_ (5%) atmosphere. Parasites cultured in the absence of compounds were used as controls of viability (negative control), while parasites exposed to chloroquine were used as positive controls. After 72 h of exposure, DNA from parasites was extracted and purified using a lysis solution containing proteinase K. Each compound and each concentration were assessed in triplicate in at least two separate experiments.

The leishmanicidal activity was tested on intracellular amastigotes of *Leishmania* *panamensis* by flow cytometry following the methodology previously described [14,15]. Previously, huMDM were infected with promastigotes of *L. panamensis* transfected with the green fluorescent protein gene (MHOM/CO/87/UA140pIR-GFP) in a proportion of 35:1 (parasites:cells). Plates were incubated at 34 °C, 5% CO_2_, and after 3 h, cells were washed twice with phosphate buffer (PBS) and incubated again. After 24 h, the infected cells were exposed for 72 h to each concentration of the compounds. Cells were removed using trypsin/EDTA solution and washed twice with PBS by centrifuging 10 min at 1,100 rpm, 4 °C. Then, cells were analyzed in a flow cytometer (Cytomics FC 500MPL, Brea, CA, USA) by reading at 488-nm excitation, 525-nm emissions over an argon laser and counting 10,000 events. The percentage of infected cells was determined by dot plot analysis, while the parasitic load was calculated by the mean fluorescence intensity using histogram analysis. In parallel, cells incubated in medium alone were used as the controls of infection (negative control), and cells exposed to amphotericin B were used as controls of leishmanicidal activity (positive control) [14,15]. Each concentration was assessed in triplicate in at least two independent experiments.

### 3.4. Statistical Analysis

Cytotoxicity was determined according to the percentages of viability and mortality obtained for each concentration and experimental condition (rotenone, derivatives, amphotericin B, chloroquine, doxorubicin, culture medium), and the results are expressed as the median lethal concentration (LC_50_) calculated by the probit analysis [21]. The percentage of viability (%V) was calculated using Equation 1, where the optical density (OD) of unexposed cells (negative control) corresponds to 100% of viability. In turn, mortality percentage corresponds to 100%–% viability:

% Viability = (OD Exposed cells)/(OD Control cells) × 100(1)

The cytotoxic activity was graded according to the LC_50_ values using the arbitrary scale: toxic, LC_50_ < 100.0 μM; moderately toxic, LC_50_ >100–< 200.0 μM; and potentially nontoxic, LC_50_ > 200 μM.

Antiplasmodial activity was determined by the inhibition of parasite growth for each experimental condition according to the florescence emitted in arbitrary units (FAU), using Equation 2, where the FAU of unexposed cells (negative control) corresponds to 100% parasite growth. In turn, % inhibition of parasite growth corresponds to 100%–% parasite growth. Results are expressed as the median effective concentration (EC_50_) calculated by probit analysis using the % of inhibition [21]:

% parasite growth = (FAU Exposed parasites)/(FAU unexposed parasites) × 100(2)

Antileishmanial activity was determined according to the reduction of infected cells and their parasite load obtained for each experimental condition according to the fluorescence median intensity (FMI) emitted by the cytometer and using Equation 3, where the % of parasitemia in unexposed cells corresponds to 100% parasitemia. In turn, the percentage of inhibition of parasitemia corresponds to 100% infection. Results are expressed as the median effective concentration (EC_50_) calculated by probit analysis using the % of inhibition of infection [21]:

% parasitemia = (FMI Exposed cells)/(FMI unexposed cells) × 100(3)

The antiplasmodial and antileishmanial activity was graded according to the CE_50_ values using the following arbitrary scale: active, EC_50_ < 20.0 μM; moderately active, EC_50_ >20–<50.0 μM; and potentially non-active, EC_50_ > 50.0 μM. The index of selectivity was calculated with the equation: IS = LC_50_/EC_50_.

## 4. Conclusions

Rotenone was selected because it is a product abundant in Nature, with a known effect on the mitochondrion of many organisms, and it has several functional groups, which can be quite easily functionalized to optimize the activity or to obtain a wider pharmacological profile. Results suggest that in spite of the toxicological background, rotenone and its derivatives have potential against malaria and cutaneous leishmania, possibly by inducing the production of ROS metabolites that are potent antiprotozoal products or modulating redox signaling in parasites. Targeting of *Plasmodium* and *Leishmania* redox metabolism may occur by modulation of redox equilibrium, damaging the mitochondrial ultrastructure or targeting essential processes and pathways with different apoptogenic agents and inhibitors [22,23,24,25,26]. We also show here that rotenone and its derivatives have differential cytotoxicity, being more cytotoxic against immortalized cell lines in cells, such as U-937, Vero, HepG2 or CAD-R1, than in cells derived from primary cultures, like huRBC and huMDM. The higher selectivity for tumor cells suggests a potential that must be confirmed in cancer cells.

Minor structural modifications in the rotenone molecule allow synthesizing a series of derivatives with antiplasmodial or antileishmanial activity that deserve further study to improve the *in vitro* activities and the selectivity of rotenone, but also its pharmacokinetic parameters. In the specific case of antileishmanial activity, only compound **6** displays activity against intracellular amastigotes, suggesting that slight structural changes may modify pharmacodynamic aspects, showing or enhancing the biological activity of one inactive compound, such as rotenone. Due to the small number of obtained compounds, a clear relationship structure-activity was not established; however, *in vitro* assays seems to point towards the importance of the exocyclic double bond and the carbonyl group to modulate the antiparasite activity.

The synthesis, antileishmanial, antiplasmodial and cytotoxicity screening of rotenone and six derivatives were reported. In spite of their toxicities, compound **6**, rotenone and derivatives **2**, **3**, **4** and **7** represent starting points in the search for new antileishmanial and antiplasmodial compounds, respectively, which would, however, require testing further structural modifications and thorough investigations on structure-activity relationships.
